# Oncolytic reovirus enhances rituximab-mediated antibody-dependent cellular cytotoxicity against chronic lymphocytic leukaemia

**DOI:** 10.1038/leu.2015.88

**Published:** 2015-04-24

**Authors:** C Parrish, G B Scott, G Migneco, K J Scott, L P Steele, E Ilett, E J West, K Hall, P J Selby, D Buchanan, A Varghese, M S Cragg, M Coffey, P Hillmen, A A Melcher, F Errington-Mais

**Affiliations:** 1 Section of Oncology and Clinical Research, Leeds Institute of Cancer and Pathology (LICAP), University of Leeds, St James's University Hospital, Leeds, UK; 2 St James's University Hospital, Leeds, UK; 3 Antibody and Vaccine Group, Cancer Sciences Unit, Southampton University, General Hospital, Southampton, UK; 4 Oncolytics Biotech Inc., Calgary, AB, Canada; 5 Experimental Haematology, LICAP, University of Leeds, Leeds, UK

## Abstract

The naturally occurring oncolytic virus (OV), reovirus, replicates in cancer cells causing direct cytotoxicity, and can activate innate and adaptive immune responses to facilitate tumour clearance. Reovirus is safe, well tolerated and currently in clinical testing for the treatment of multiple myeloma, in combination with dexamethasone/carfilzomib. Activation of natural killer (NK) cells has been observed after systemic delivery of reovirus to cancer patients; however, the ability of OV to potentiate NK cell-mediated antibody-dependent cellular cytotoxicity (ADCC) is unexplored. This study elucidates the potential of oncolytic reovirus for the treatment of chronic lymphocytic leukaemia (CLL), both as a direct cytotoxic agent and as an immunomodulator. We demonstrate that reovirus: (i) is directly cytotoxic against CLL, which requires replication-competent virus; (ii) phenotypically and functionally activates patient NK cells via a monocyte-derived interferon-α (IFNα)-dependent mechanism; and (iii) enhances ADCC-mediated killing of CLL in combination with anti-CD20 antibodies. Our data provide strong preclinical evidence to support the use of reovirus in combination with anti-CD20 immunotherapy for the treatment of CLL.

## Introduction

Chronic lymphocytic leukaemia (CLL) is the most common form of adult leukaemia in the western world and is characterised by the accumulation of CD19^+^CD5^+^ malignant B lymphocytes in the blood, bone marrow and secondary lymphoid organs. Disease stage and chromosomal aberrations are recognised to have prognostic value, and lower levels of circulating T/natural killer (NK) cells have also been reported to confer a poor prognosis, suggesting a contribution of immune-mediated tumour regulation.^1^

Survival from diagnosis ranges from only months to decades and therapy is increasingly tailored to both disease and patient factors, in particular, patients' fitness and their ability to tolerate treatment toxicity. The chimeric monoclonal antibody, rituximab, targets CD20, an antigen expressed on both normal and malignant B cells, but absent from B-cell precursors, mature plasma cells and non-lymphoid tissues.^2^ Rituximab has activity against CLL as a monotherapy, but particularly impacts on prognosis when used in combination with chemotherapy, for example, with fludarabine and cyclophosphamide, where significant response rates are seen in both untreated and heavily pretreated patients (complete remission in ~50% of patients). Despite such advances, CLL remains incurable and the clinical course is characterised by persistent minimal residual disease and the acquisition of mutations conferring drug resistance.^3, 4^ Much of the recent focus in CLL has been on targeting B-cell receptor and chemokine signalling pathways, but as potent as these agents appear, drug resistance is nonetheless emerging.^4^ It is therefore critical that the anticancer armamentarium continues to expand, focussing on targeted, low-toxicity therapies with distinct mechanisms of action, which can be used in combination with existing and novel agents to overcome minimal residual disease.

The activity of rituximab against B-cell malignancies is mediated via several mechanisms including antibody-dependent cellular cytotoxicity (ADCC) and complement-dependent cytotoxicity.^5^ Rituximab-mediated ADCC, encompassing antibody-dependent cellular phagocytosis, is well characterised and roles for monocytes, macrophages and NK cells have been described.^6^ Strategies have been investigated to increase the efficacy of rituximab-mediated ADCC, such as disruption of killer inhibitory receptors on NK cells, or immune activation using the immunomodulatory agent, lenalidomide.^7, 8^ Second- and third-generation anti-CD20 antibodies, with altered modes of action, are also under clinical investigation,^2^ including ofatumumab (which induces more potent complement-dependent cytotoxicity),^9^ and obinuntuzumab (GA101), which has a glyco-engineered Fc portion for enhanced ADCC.^10^

Oncolytic viruses (OVs) are currently being investigated for the treatment of a range of solid malignancies and there is increasing clinical evidence supporting their safety and efficacy, both as a monotherapy and in combination with chemotherapy or radiotherapy.^11, 12^ Preclinical evidence supporting clinical trial development for OV in haematological malignancies remains limited.^13, 14, 15^ Reovirus is a naturally occurring double-stranded RNA virus, which exerts its anticancer effects by direct oncolysis and activation of antitumour immunity.^16^ Reovirus activation of NK cells, *in vitro*, can increase lysis of tumour cell targets^17^ and has been observed after systemic delivery to cancer patients.^18^ Previous literature has suggested that NK cell function is defective in CLL patients, due to decreased production of cytolytic granules^19^ and inhibitory 4-1BB/4-1BBL interactions with malignant CLL cells.^20^

This study investigates the efficacy of reovirus for the treatment of CLL, both as a direct cytotoxic agent and as an immunomodulator, and is the first study to examine activation of NK cells by OV in the context of CLL. The data presented clearly illustrate the direct cytotoxic and immunomodulatory potential of reovirus, and provide strong evidence to support the development of early-stage clinical trials to evaluate reovirus-based combination therapy in patients with CLL.

## Materials and methods

### Cell culture and reagents

Human CLL cell lines were purchased from German Collection of Microorganisms and Cell Cultures (DSMZ) and were authenticated to confirm they are a perfect match to the DSMZ database. EHEB cells were grown in RPMI (Sigma-Aldrich, St Louis, MO, USA) supplemented with 10% (v/v) foetal calf serum (FCS; Life Technologies, Grand Island, NY, USA) and MEC-2 cells were grown in Iscove's modified Dulbecco's medium (Sigma-Aldrich), 20% (v/v) FCS. Murine L929 cells were purchased from American Type Culture Collection (Middlesex, UK) and grown in Dulbecco's modified Eagle's medium (Sigma-Aldrich) containing 10% (v/v) FCS. Routine tests ensured that all cell lines were mycoplasma free.

Peripheral blood mononuclear cells (PBMCs) were isolated from CLL patients after written, informed consent was obtained in accordance with local institutional ethical approval. PBMCs were isolated from whole blood by density gradient centrifugation on Lymphoprep (Axis-Shield, Oslo, Norway). Freshly isolated PBMCs were cultured at a high cell density (5 × 10^6^ cells per ml) in RPMI supplemented with 10% (v/v) FCS, as previously described by Willmore *et al.*^21^ Primary CLL samples were cultured as whole PBMCs immediately after collection, without freezing cells down or isolating CLL cells from the rest of the PBMCs, which significantly reduced their viability. We postulate that the enhanced viability using this culture method is because of the presence of nurse-like cells/CD14^+^ monocytes, which maintain CLL viability—in support of this, we observed a marked loss of CLL viability after removal of CD14^+^ monocytes from this culture system (data not shown). To investigate direct cytotoxicity, patients with high white blood cell counts were used (>25 × 10^9^/l), and for immune focused experiments, patients with lower white blood cell counts were used (<25 × 10^9^/l). Patient characteristics are outlined in Table 1.

Reovirus type 3 dearing strain (Reolysin) was provided by Oncolytic Biotech Inc. (Calgary, AB, Canada) and virus titre was determined by standard plaque assay on L929 cells. For UV inactivation, a Stratalinker UV 1800 Crosslinker (Stratagene, La Jolla, CA, USA) was used and loss of viral replication was confirmed by plaque assays. Rituximab (MabThera; Roche, Welwyn Garden City, UK) was purchased from St James's University Hospital (Leeds, UK). Ofatumumab and GA101 were generated in-house as previously described from patent published sequences.^22^

#### Reovirus treatment

Patient PBMCs were cultured at 37 °C in a humidified atmosphere and either left untreated or treated with replication-competent or UV-inactivated reovirus, at stated multiplicities of infection (MOIs). Different MOIs were used for direct cytotoxicity assays and immune studies to reflect likely deliverable tissue doses *in vivo*. Lower MOI (0.1 and 1), which are achievable and deliverable to patients, were used for NK cell experiments as NK cells should be activated almost immediately after intravenous delivery, in the absence of viral replication. In contrast, direct cytotoxicity is associated with *in situ* viral replication and as such CLL cells are more likely to be exposed to higher MOI (1 and 10), at prolonged time points after infection.

### Cell viability

Cells were harvested, washed in FACS buffer (phosphate-buffered saline (Sigma-Aldrich) containing 1% (v/v) FCS and 1% (v/v) sodium azide (Sigma-Aldrich)) and cell viability determined by propidium iodide (PI, 0.05 mg/ml; Sigma-Aldrich) staining.

### Reovirus replication

Cells were treated with 1 plaque-forming unit (PFU) per cell reovirus, cells and supernatants were harvested and subjected to three rounds of freeze–thaw using a 37 °C water bath and methanol/dry ice. Fold increase in virus titre was determined by comparison with input virus using plaque assay on L929 cells.

### Flow cytometry

Flow cytometry was performed using FACSCalibur (BD Biosciences, Oxford, UK) and Attune (Life Technologies) flow cytometers, and data were analysed using Cell Quest Pro (BD Bioscience) and Attune Cytometric software v.2.1 (Life Technologies).

#### Surface phenotyping

NK cells were identified as CD3^−^CD56^+^ and CLL cells as CD19^+^CD5^+^. Expression of CD69 on NK cells, and JAM-1, HLA-Class I, ULBP-1, ULBP-2, MICA/B, CD112, CD155 and CD20 on CLL cells, was determined by flow cytometry. Antibodies were purchased from BD Biosciences (anti-CD69 FITC (fluorescein isothiocyanate), anti-HLA-Class I PE, anti-MICA/B PE, anti-CD112 PE, anti-CD3 PerCP and anti-CD19 FITC), R&D Systems (Minneapolis, MN, USA; anti-ULBP-1 and -2 PE and anti-CD155 PE), AbD Serotec (Kidlington, UK; anti-CD56 PE), Life Technologies (anti-CD5 APC and anti-CD20 PE) and Millipore (Billerica, MA, USA; anti-JAM-1 PE). To examine the surface binding of rituximab, CLL cells were labelled for 30 min (10^6^ cells per ml) with different concentrations of rituximab (at 37 °C), washed in FACS buffer and counterstained with FITC-labelled mouse anti-human IgG antibody (BD Biosciences).

#### CD107a/b cytotoxicity assay

CLL patient PBMCs were treated with reovirus overnight and cocultured with EHEB, MEC-2 or autologous CLL targets (±rituximab or isotype control (R&D Systems)) at a 10:1 effector:target ratio. Cells were cultured for 1 h before the addition of anti-CD3 PerCP and anti-CD107a/b FITC (BD Biosciences), anti-CD56 PE (Miltenyi Biotec, San Diego, CA, USA) and 10 μg/ml brefeldin A (BioLegend, San Diego, CA, USA) for 4 h. Expression of CD107a/b on CD3^−^CD56^+^ NK cells was determined.

#### Intracellular IFNα

For intracellular IFNα detection, whole PBMCs, CD14^+^ monocyte-depleted PBMCs or isolated CD14^+^ monocytes (MACS CD14 positive selection; Miltenyi Biotech) were used. Isolated monocyte preparations were >90% pure, CD14^+^-depleted PBMCs contained >93% lymphocytes, compared with 80% lymphocytes in unselected PBMC populations. Cells were subsequently treated with reovirus for 16 h before the addition of 10 μg/ml brefeldin A for a further 5 h. Cells were washed in FACS buffer, permeabilised in 0.3% saponin and stained with anti-IFNα PE (BD Biosciences) for 1 h in 0.1% saponin, before flow cytometry.

### ^51^Chromium release assay

PBMCs from healthy donors or CLL patients were activated with reovirus for 16 h and their ability to lyse CLL cell targets was determined using a 4 h ^51^Chromium (^51^Cr) release assay. Briefly, PBMCs were seeded at different effector:target ratios and cocultured with ^51^Cr (Perkin-Elmer, Waltham, MA, USA)-labelled EHEB/MEC-2 cell targets (±rituximab or isotype control) for 4 h. Cells were pelleted by centrifugation and 50 μl of supernatant was transferred onto lumaplates (Perkin-Elmer) before analysis using a Wallac Jet 1459 Microbeta scintillation counter and Microbeta software (Perkin-Elmer). Percent lysis was calculated using the following formula: % lysis=100 × (sample c.p.m.−spontaneous c.p.m.)/(maximum c.p.m.−spontaneous c.p.m.).

### IFNα neutralisation and detection

To neutralise type I IFNs, PBMCs were treated with reovirus for 16 h±neutralising antibodies (NAbs; IFN block; PBL Interferon Source, Piscataway, NJ, USA) or isotype control (R&D Systems). IFN block consisted of sheep polyclonal antibodies (0.75% of anti-IFN-α and -β) and mouse monoclonal anti-human IFN-α/β receptor chain 2 (1.25%). For isotype controls, 1.5% (v/v) sheep serum (Sigma-Aldrich) and 1.25% (v/v) mouse IgG2a was used. Secretion of IFNα from PBMCs (±reovirus) was determined by enzyme-linked immunosorbent assay (ELISA) using matched-pair antibodies (Mabtech, Cincinnati, OH USA).

### Statistics

Statistical analysis was performed using GraphPad Prism software (GraphPad, La Jolla, CA, USA) using paired *t*-tests, and one- and two-way analysis of variance, as appropriate. Levels of statistical significance: *P⩽0.05, **P⩽0.01, ***P⩽0.001, ****^/†^*P*⩽0.0001.

## Results

### CLL cells are susceptible to reovirus-mediated oncolysis

**Figure 1:**
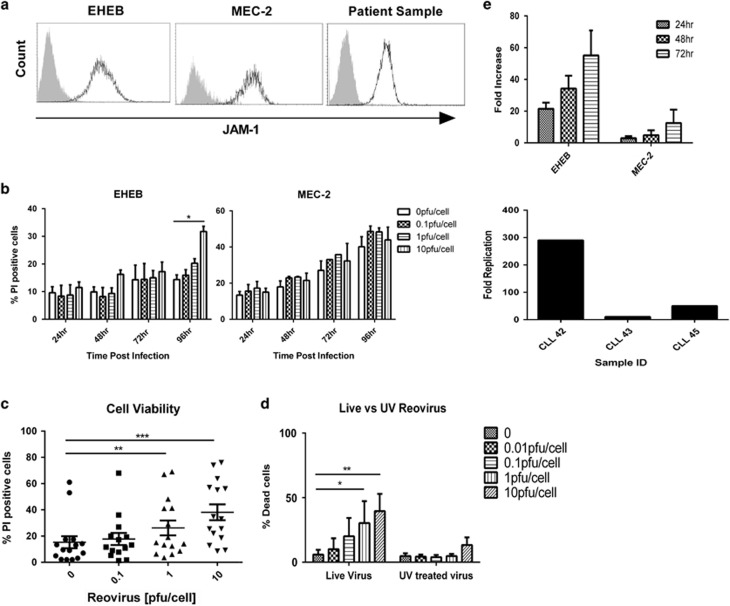
Direct cytotoxic effects of reovirus against CLL. (**a**) JAM-1 expression on EHEB and MEC-2 CLL cell lines (*n*=2) and primary CLL samples (*n*=4). Histogram overlay plots show representative flow cytometry data (shaded area=isotype; black line=JAM-1). (**b**) EHEB and MEC-2 CLL cell lines were treated with different doses of reovirus and cell viability was determined by PI staining at 24, 48, 72 and 96 h after treatment. Bar charts show the mean percentage of PI^+^ cells (*n*=3, ±s.e.m.). (**c**) Primary CLL samples were treated with reovirus for 7 days and cell viability was determined by PI staining (*n*=15, ±s.e.m.). (**d**) Primary CLL samples were treated with live or UV-inactivated reovirus for 7 days and the percentage of PI^+^ cells was determined. Data shown are the mean of *n*=4 independent experiments (±s.e.m.). (**e**) Reovirus replication was determined in EHEB and MEC-2 cell lines (24, 48 and 72 h after infection) and primary CLL samples (7 days after infection). Reovirus concentration was determined by plaque assay and the fold increase in viral titre was determined by comparison with input virus. Data for EHEB and MEC-2 cells is the mean (*n*=3, ±s.e.m.), and data from three primary samples where viral replication was observed are shown.

To investigate the potential of reovirus for the treatment of CLL cells, the expression of the reovirus receptor (JAM-1) on CLL cell lines (EHEB and MEC-2) and primary CLL cells from patients was examined; EHEB/MEC-2 cell lines and all primary samples expressed JAM-1 (Figure 1a; *n*=4). Next, the direct cytotoxic effect of reovirus was determined by PI staining: EHEB and MEC-2 cell lines were treated with reovirus for 24, 48, 72 and 96 h and the percentage of dead cells at each time point is illustrated in Figure 1b. The cell lines were relatively resistant to reovirus-induced death, with only a small increase in cell death seen in EHEB at 96 h (~20%) and no significant cytotoxicity against MEC-2. To confirm this observation, which was unexpected in light of previous reports,^13^ primary CLL samples (*n*=15) were treated with reovirus for 7 days. Toxicity was observed over this prolonged period of infection (Figure 1c), although not at earlier time points (data not shown); these data suggest that primary CLL cells are susceptible to reovirus-induced death, although significant patient variation was observed. Use of a gating strategy to exclude cell aggregates/doublets, before quantification of PI^+^ cells, confirmed that reovirus does not cause significant cell aggregation (in keeping with our previous experience); hence, the reovirus-induced toxicity illustrated in Figure 1c was not an artefact of aggregate formation (Supplementary Figure 1).

To determine whether reovirus replication was required for the cytotoxic effects observed, primary CLL cells were treated with live or UV-inactivated virus for 7 days and cell viability was examined; cytotoxicity was dependent on replication competence (Figure 1d). Reovirus replication was also confirmed in susceptible EHEB cells, and to a lower degree in reovirus-resistant MEC-2 cells (Figure 1e); western blot analysis for the expression of the reovirus sigma 3 protein, at 0, 3, 24, 48, 27 and 96 h after infection, confirmed reovirus protein production in both the EHEB and MEC-2 cells, with elevated levels in the EHEB cells at earlier time points (Supplementary Figure 2). These data corroborate the plaque assay data and suggest that the increased sensitivity of EHEB cells (Figure 1b) is because of increased reovirus replication. Plaque assays confirmed reovirus replication in primary CLL patient samples, which was detectable in three of eight patient samples (Figure 1e).

Reovirus stimulates inflammatory cytokine production from tumour cells and immune effectors,^23, 24, 25^ which may potentially support CLL proliferation.^26^ To examine this, patient PBMCs were treated with reovirus for 7 days and expression of Ki-67 was determined; as a positive control, CLL cells were stimulated with αCD3/αCD28 microbeads to activate T cells and stimulate CLL proliferation.^26^ Reovirus did not increase the percentage of Ki-67^+^ cells or CLL proliferation (Supplementary Figure 3).

#### Reovirus activates CLL patient NK cells and stimulates innate antitumour immunity

**Figure 2:**
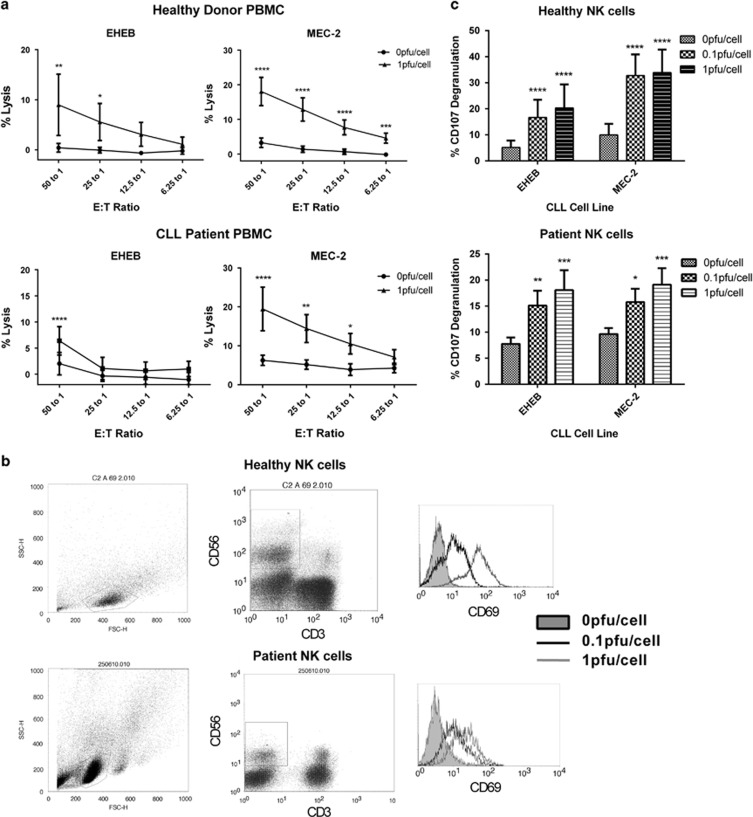
Cytotoxicity of reovirus-activated PBMCs against CLL. Healthy donor PBMCs and CLL patient PBMCs were isolated and either left untreated, or treated with 0.1 or 1 PFU/cell reovirus for at least 16 h. (**a**) PBMCs were used in a 4 h ^51^Cr release assay against EHEB and MEC-2 CLL cell targets, at different effector:target (E:T) ratios. Line graphs show the mean (±s.e.m.) % lysis for healthy donors (*n*=3) and patient samples (*n*=6). (**b**) Expression of CD69 (an early activation marker) on CD3^−^/CD56^+^ NK cells was determined. Histogram plots show representative data for CD69 expression±reovirus treatment. Representative data are shown for healthy donors (*n*=3) and patient samples (*n*=4). (**c**) PBMCs from healthy donors or patient samples (±reovirus) were cocultured with EHEB or MEC-2 cell targets and the expression of CD107a/b (a marker of cytotoxic granule release) on CD3^−^CD56^+^ NK cells was determined. Bar charts show the mean percentage (±s.e.m.) of total NK cells expressing CD107a/b for healthy donors (*n*=4) and patient samples (*n*=6).

It is well documented that reovirus can exert its antitumour effects by direct oncolysis or activation of antitumour immunity,^24, 25, 27^ hence the ability of reovirus to stimulate immune-mediated killing of CLL was investigated. PBMCs from healthy donors or CLL patients were isolated and treated with 0, 0.1 or 1 PFU/cell reovirus for 16 h. In these experiments, PBMCs, rather than isolated cell populations, were used to allow cellular interactions that might initiate immune-mediated cytotoxicity against malignant B cells. Reovirus activated PBMCs to kill CLL EHEB/MEC-2 cell targets, as determined by ^51^Cr release assay (Figure 2a).

NK cells are key innate immune effectors known to be important for the efficacy of rituximab- and lenalidomide-based immunotherapy for CLL.^8, 28^ To investigate the role of NK cells, PBMCs from healthy donors and patient samples were incubated with reovirus and NK cell (CD3^−^CD56^+^) activation was determined by CD69 and CD107a/b surface expression (Figures 2b and c, respectively). CD69 upregulation was observed after treatment with 0.1 and 1 PFU/cell reovirus in both healthy donor and patient samples, which correlated with increased NK cell degranulation upon coculture with EHEB and MEC-2 cell targets. Expression of NKG2D, DNAM-1, NKp30, NKp44 and NKp46 on donor–patient NK cells was also examined and no reproducible changes were observed after reovirus treatment (data not shown).

**Figure 3:**
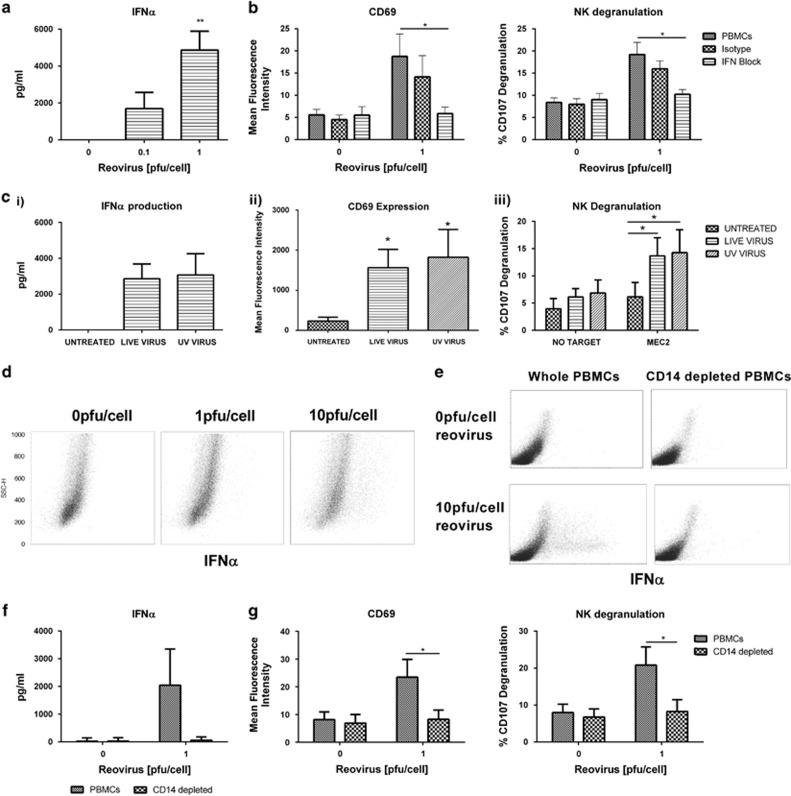
Mechanism of NK cell activation by reovirus in CLL patient samples. (**a**) PBMCs from CLL patients were treated with reovirus, cell-free supernatant was collected and the secretion of IFNα was determined by ELISA. Bar chart shows mean (*n*=11, ±s.e.m.). (**b**) CLL patient PBMCs were either left untreated or treated with 1 PFU/cell reovirus overnight, in the presence or absence of type 1 IFN-blocking antibodies or appropriate isotype controls. Bar charts show the mean (*n*=3, ±s.e.m.) NK cell activation as determined by CD69 expression or CD107a/b degranulation against MEC-2 targets. (**c**) PBMCs were treated with replication-competent (live) or UV-inactivated reovirus and (i) the production of IFNα was determined by ELISA 24 h after treatment (*n*=3, ±s.em.), (ii) the expression of CD69 on NK cells (*n*=4, ±s.e.m.) and (iii) levels of CD107 NK cell degranulation were determined (*n*=4, ±s.e.m.). (**d**) CD14^+^ monocytes were isolated from CLL samples and left overnight to allow CD14 microbeads to dissociate; monocytes were treated with reovirus and IFNα expression was determined by intracellular flow cytometry (representative of *n*=2). (**e**–**g**) PBMCs from CLL patient samples were either left untreated or treated with reovirus, ±CD14^+^ monocyte depletion. (**e**) Intracellular IFNα was determined by flow cytometry (representative o f *n*=2). (**f**) Levels of IFNα secretion were determined by ELISA (*n*=5, ±s.e.m.). (**g**) NK cell activation was determined by CD69 expression or CD107a/b degranulation (*n*=6, ±s.e.m.).

Next, to explore the mechanism of NK cell activation, we hypothesised that reovirus-induced type 1 IFNs may be responsible for the activation of NK cells. Figure 3a demonstrates the secretion of IFNα, but not IFNβ (data not shown), by CLL patient PBMCs after reovirus treatment. Moreover, blocking type 1 IFNs during reovirus treatment completely abrogated NK cell activation, preventing upregulation of CD69 and degranulation (Figure 3b); addition of recombinant IFNα was also sufficient to activate NK cells (CD69 upregulation and NK cell degranulation) to a level comparable to that observed after reovirus treatment (Supplementary Figure 4). Furthermore, we have confirmed that NK cell activation occurs independently of reovirus replication as IFNα production (Figure 3ci), NK cell CD69 upregulation (Figure 3cii) and NK cell degranulation (Figure 3ciii) are equivalent with replication-competent or UV-inactivated virus (Figure 3c). To determine the cell type responsible for IFN secretion in response to reovirus, isolated monocytes (which can produce IFNα in response to poly I:C)^29^ were positive for intracellular IFNα after reovirus treatment (Figure 3d). Removal of CD14^+^ cells from PBMCs abolished IFNα-positive cells by intracellular flow cytometry (Figure 3e), and prevented detection of IFNα by ELISA (Figure 3f). CD14^+^ depletion also prevented increased NK cell CD69 expression and degranulation (Figure 3g), confirming that monocytes are essential for NK cell activation; however, this does not preclude the involvement of other cell populations within the mixed PBMC population.

#### Potentiating ADCC using combination viroimmunotherapy

**Figure 4:**
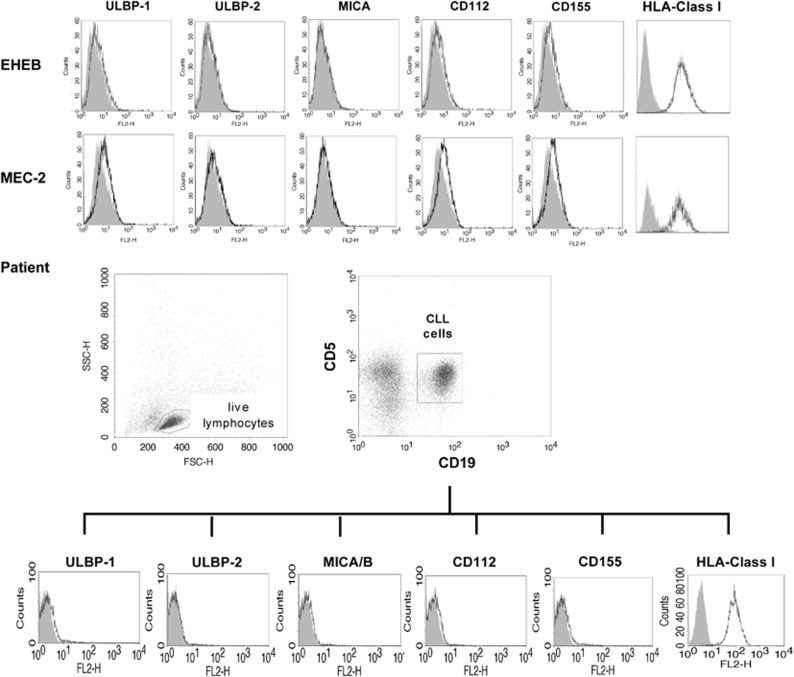
Expression of NK cell activatory ligands on CLL cells. Expression of the NK activatory ligands ULBP-1, ULBP-2, MICA/B, CD112, CD155 and HLA-Class I on EHEB, MEC-2 and patient CLL cells was determined by flow cytometry. CLL cells within patient PBMCs were selected using a double-gating strategy, gating on lymphocytes followed by CD19^+^CD5^+^ cells. Data shown are representative of *n*=4 independent experiments.

Although NK cell degranulation was observed after reovirus activation of PBMCs, the levels of CLL tumour cell lysis achieved were low, with only ~10% killing of EHEB and ~20% killing of MEC-2 (Figure 2a). We considered whether the levels of NK cell activatory ligands, and HLA-Class I, on CLL cells may contribute to this low level of killing. These studies confirmed that expression of the ligands for NKG2D (ULBP-1, ULBP-2 and MICA/B) and DNAM-1 (CD112 and CD155) on CLL cell lines and primary samples was low/absent; in contrast, significant HLA-Class I expression was observed (Figure 4). Preliminary investigations demonstrated that reovirus did not alter NK cell ligand expression (data not shown).

**Figure 5:**
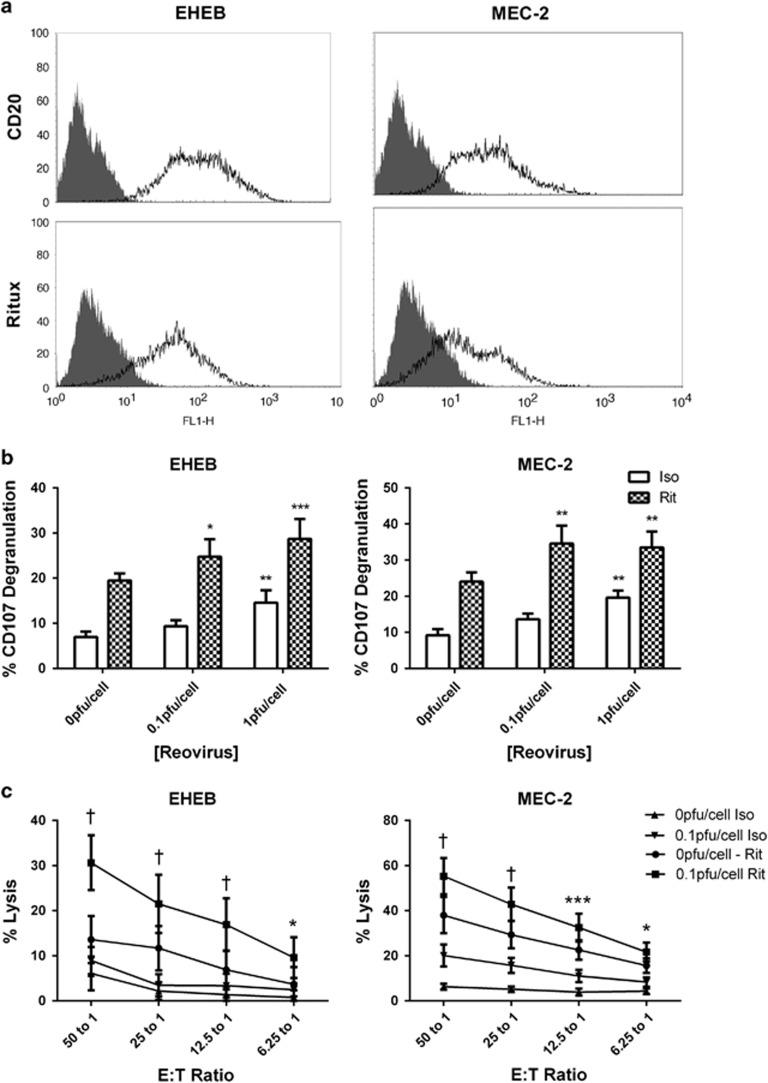
Reovirus potentiates rituximab-mediated ADCC. (**a**) Representative histogram plots showing the expression of CD20, and rituximab binding, on EHEB and MEC-2 CLL cells are shown (*n*=2–3). (**b**) PBMCs from CLL patient samples were either left untreated or cultured overnight with reovirus. NK cell (CD3^−^CD56^+^) CD107a/b degranulation was determined after coculture with rituximab- or isotype control-labelled EHEB or MEC-2 cell targets. Bar charts show the mean percentage of total NK cells expressing CD107a/b (*n*⩾6, ±s.e.m.). (**c**) Four-hour ^51^Cr release assays were carried out using patient PBMCs that were either left untreated or activated with reovirus overnight, and cocultured with rituximab- or isotype control-labelled EHEB or MEC-2 cell targets. Line graphs compare the mean % lysis (*n*=6± s.e.m.) between 0  and 0.1 PFU/cell. Statistical significance between 0 vs 0.1 PFU/cell for rituximab-labelled targets is shown.

Nevertheless, as reovirus activation of NK cells enhanced killing of CLL cells, albeit at a low level, we investigated the addition of rituximab (which is thought to act in part via NK cell-mediated ADCC) to reovirus-treated PBMCs. Expression of CD20 on EHEB and MEC-2 cells, and their ability to bind rituximab, was first confirmed (Figure 5a). Next, the ability of NK cells from CLL patient samples to degranulate against rituximab-opsonised cells (±reovirus stimulation) was investigated. Rituximab labelling of CLL cells alone increased PBMC NK cell degranulation; however, reovirus treatment significantly increased NK cell CD107a/b degranulation compared with rituximab alone (Figure 5b). ^51^Cr release assays confirmed that increased NK cell degranulation, after combination treatment with reovirus and rituximab, correlated with enhanced lysis of CLL cell targets (Figure 5c). In particular, killing of EHEB cells, which were relatively resistant to NK lysis after reovirus activation, was markedly improved using the combination. Increased killing of isotype-labelled control cells was observed after reovirus treatment, in the absence of rituximab, which correlates with results shown in Figure 2. Similar results were also observed using (i) healthy donor PBMCs as effectors, rather than CLL patient PBMCs (Supplementary Figure 5), and (ii) UV-inactivated reovirus or recombinant IFNα to activate NK cells and potentiate rituximab-mediated ADCC (Supplementary Figure 6).

**Figure 6:**
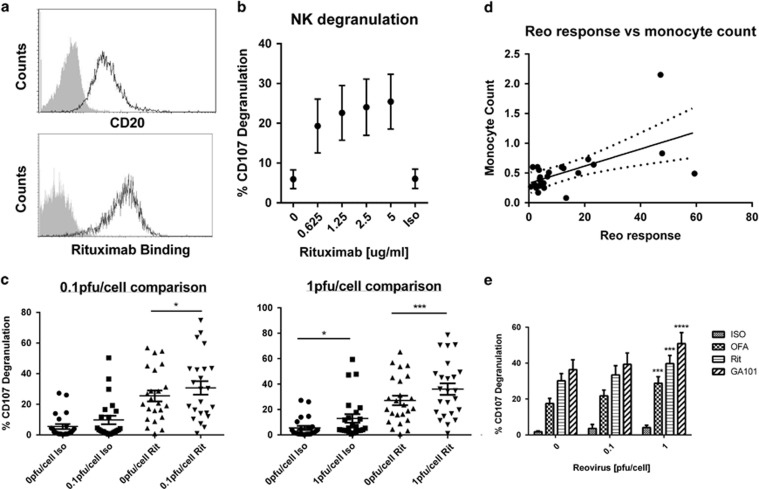
Reovirus enhances NK cell ADCC activity against rituximab-labelled autologous CLL cell targets. (**a**) Expression of CD20 and rituximab binding to CLL cells within patient PBMCs. CLL cells were identified as described in Figure 4 and data shown is representative of *n*=3 patient samples. (**b**) Patient PBMCs/CLL cells were labelled with increasing doses of rituximab or isotype control and cocultured with autologous PBMCs. CD3^−^CD56^+^ NK cell CD107a/b degranulation was determined and scatter plots show the mean percentage of total NK cells expressing CD107a/b (*n*=12, ±s.e.m.). (**c**) Patient PBMCs were either left untreated or activated with reovirus overnight and cocultured with rituximab/isotype-labelled autologous CLL cells. Scatter plots show CD3^−^CD56^+^ NK cell CD107a/b degranulation for each individual sample (*n*=24), mean (±s.e.m.) and comparison of 0 PFU/cell vs 0.1 and 0 vs 1 PFU/cell. (**d**) Linear regression analysis of reovirus-induced NK cell activation (NK cell degranulation after treatment with 1 PFU/cell reovirus overnight, *n*=24) and absolute monocyte count (×10^9^/l) demonstrating a significant correlation (*P*=0.0023). (**e**) Comparison of CD3−CD56^+^ NK cell CD107a/b degranulation after coculture with isotype control-, ofatumumab-, rituximab- and GA101-labelled autologous CLL cells, ±reovirus activation of PBMCs, is shown (*n*=7).

NK cells were activated by reovirus and this activation was not impeded by the presence of malignant CLL cells. However, it was important to address the potential of the reovirus/rituximab combination approach in a fully autologous system. CLL cells from patient samples express CD20 and bind rituximab (Figure 6a). A dose-dependent increase in NK cell degranulation against rituximab-labelled autologous CLL cells was observed (Figure 6b); 5 μg/ml rituximab was chosen for subsequent experiments. Patient samples (*n*=24) were then evaluated to test the therapeutic potential of combining reovirus with rituximab to potentiate NK cell-mediated ADCC of rituximab-labelled CLL cells. NK cell degranulation against autologous CLL cells was increased upon coculture with rituximab-labelled cells (0 PFU/cell Rit) or activation with reovirus alone (1 PFU per cell Iso); however, the combination of reovirus (0.1 or 1 PFU/cell) with rituximab significantly further increased NK cell degranulation compared with either treatment alone (Figure 6c). Interestingly, one patient (used for NK cell degranulation) had received prior rituximab treatment. This patient continued to have weak CD20 expression and NK cell degranulation was augmented in the presence of reovirus.

Approximately 25% of the 24 patient samples did not respond to reovirus treatment (i.e. only a small increase in NK cell degranulation was seen). As monocytes were required for NK cell activation, we examined whether the response to reovirus correlated with patient absolute monocyte count (×10^9^/l). Figure 6d shows linear regression analysis of NK cell degranulation in response to 1 PFU/cell reovirus *vs* patient absolute monocyte count, this confirmed a positive correlation between reovirus-induced NK cell activation and peripheral blood monocyte levels (*P*=0.0023); a positive correlation was also observed between reovirus/rituximab treatment and patient absolute monocyte count (*P*=0.0186, data not shown). In contrast, response to rituximab did not correlate with monocytes levels, and NK cell activation by reovirus did not correlate with total white blood cell count (data not shown). Furthermore, when IFNα production from the same samples was determined, those that responded to reovirus treatment (i.e. increased NK cell degranulation) had greater IFNα production (>2500 pg/ml) than non-responders (<1000 pg/ml) (Supplementary Figure 7). Monocyte counts and type I IFNα production may therefore be useful predictors of NK cell activation in response to reovirus treatment.

Although rituximab is a well-established therapy for CLL, other anti-CD20 antibodies are in clinical development. To examine whether the effects observed after reovirus/rituximab could be translated to other anti-CD20 antibodies, where NK cells also have a role, rituximab was compared with ofatumumab (which stimulates more potent complement-dependent cytotoxicity^6^) and GA101 (which was engineered for enhanced ADCC effector function).^30^ Consistent with a paradigm in which reovirus enhances NK ADCC effector function, all combinations enhanced NK cell degranulation against autologous targets, with GA101/reovirus being most effective, followed by rituximab/reovirus, and then the ofatumumab/reovirus combination (Figure 6e).

## Discussion

Although previous reports have demonstrated that CLL cells are susceptible to direct OV-mediated oncolysis,^13, 31^ data on the efficacy of reovirus or other OV for this disease are limited. Moreover, the immune-mediated therapeutic potential of reovirus in CLL has not been addressed. Tumilasci *et al.*^32^ and Samuel *et al.*^33^ reported that CLL cells were resistant to another RNA OV, vesicular stomatitis virus (VSV), owing to overexpression of Bcl-2, and inhibition of Bcl-2 rendered cells sensitive to VSV oncolysis. Our work has confirmed previous studies and demonstrated that CLL cells are susceptible to reovirus-induced oncolysis,^13^ although clear differences in patient susceptibility were observed. Reovirus induces caspase-dependent apoptosis,^23^ hence interpatient variation in pro- *vs* antiapoptotic proteins, such as Bcl-2/Bcl-xL/Bcl-w/NOXA/Mcl-1, is likely to influence virus sensitivity. Importantly, the effect of reovirus on non-leukaemic, haematopoietic stem cells has been previously described and no reovirus-induced toxicity was observed.^31^ The limited sensitivity of CLL cells to direct viral oncolysis has likely hampered preclinical development of oncolytic virotherapy in this setting. However, with increasing awareness of the immune-mediated component of oncolytic virotherapy, it is now appropriate to reconsider the immune potential of OV in the treatment of CLL.^20, 30, 34^

A small number of conflicting studies have examined NK cell phenotype and function in CLL patients, with some suggesting NK cell function is defective^19, 35^ and others showing its restoration upon cytokine activation.^36, 37^ Often these studies focused on isolated NK cells, ignoring the importance of the multiple cell-to-cell interactions between immune effectors, as well as the impact of tumour burden. In contrast, we have examined NK cell function in the context of PBMCs and demonstrated conserved NK cell function in CLL patient samples.

There is an increasing body of evidence supporting the clinical efficacy of OV for solid malignancies^12^ and preliminary data on multiple myeloma are also encouraging.^38^ It is also becoming clear that the efficacy of OV is dependent on the generation of systemic antitumour immunity, as demonstrated in numerous preclinical *in vivo* models,^27, 34, 39, 40^ and, for example, a phase II trial showing regression of distant metastatic lesions following intratumoural injection of a herpes simplex virus OV expressing granulocyte–macrophage colony-stimulating factor (GM-CSF).^41^ Although systemic delivery of OV for solid malignancies may be problematic, haematological malignancies are ideally suited to systemic viral delivery.

As reovirus is a naturally occurring double-stranded RNA virus, to which most people have been exposed during childhood, circulating NAbs are almost universal, and rise significantly after intratumoural^42^ or systemic treatment;^43^ such NAbs in the blood could potentially impact on reovirus delivery to the tumour and its ability to activate NK cells *in vivo*. However, recent studies from our group have demonstrated that NAbs do not prevent NK cell activation either *in vitro*^17^ or *in vivo*.^18^ Furthermore, reovirus delivery to colorectal liver metastases, metastatic melanoma and bone marrow-resident myeloma cells has been reported after intravenous delivery, despite increasing NAbs levels.^43, 44, 45^ Moreover, it has recently been postulated that reovirus is protected from high levels of NAbs by association with PBMCs/granulocytes.^43, 46^ Most significant is the recent unexpected observation that clearance of B16 murine melanoma after GM-CSF/reovirus treatment was actually dependent on the presence of anti-reovirus NAbs, monocytes and NK cells.^47^ Although NAbs may impede the clinical efficacy of OVs in general, clinical efficacy of reovirus has been reported when used in combination with current standard-of-care chemotherapy—a precedent therefore exists for the use of combination regimens to increase the *in vivo* efficacy of reovirus in subsequent clinical translation studies.^43, 48^

The current study suggests absolute monocyte count and the type 1 IFNα response could be used to predict the generation of antitumour innate immunity by reovirus. In our series of 24 patients, ~25% did not respond to reovirus (i.e. NK cell activation was not observed); when IFNα production was examined, failure to produce IFNα and inability of NK cells to respond were associated (Supplementary Figure 7). Furthermore, NK cell activation also correlated with absolute monocyte count. A role for IFNα is further supported by identification of an IFN gene signature within NK cells from reovirus-treated patients.^18^ Larger numbers of patients, ideally within the context of a formal clinical trial with therapeutic end points, will be needed to confirm any potential use of monocyte count as a biomarker for oncolytic immunotherapy for CLL.

As IFNα has been reported to decrease the number of CLL cells in the circulation at early disease stages (A and B), its production after reovirus treatment could generate significant bystander effects by direct cytotoxicity as well as activation of antitumour immunity.^49, 50^ Cytokine production (interleukin-6 and tumour growth factor-β) from malignant CLL cells can inhibit T and NK cells,^51, 52, 53^ and PD-1/PD-1L expression may also inhibit immune cell functions;^54^ future work will elucidate the effect of reovirus-induced IFNα production on these suppressive mechanisms.

The exact mechanism of rituximab cytotoxicity *in vivo* remains unclear. ADCC is thought to have a major part in the anticancer effects of rituximab and both NK cells (via FcγR IIIa/CD16a engagement and perforin/granzyme-mediated lysis) and myeloid cells, such as monocytes and macrophages (via engagement of various FcγR and phagocytosis), can be effectors for ADCC/antibody-dependent cellular phagocytosis.^55, 56^ Recently, a role for neutrophils has also been implicated for non-fucosylated anti-CD20 antibodies.^57^ The ability of reovirus to influence monocyte/macrophage and neutrophil effector function, and enhance ADCC/antibody-dependent cellular phagocytosis, is currently under investigation.

Currently, other strategies to harness and augment antitumour immunity are also under appraisal, for example, combination therapy with rituximab and lenalidomide (an immunomodulatory agent that enhances NK cell and monocyte function^8^ and has single-agent efficacy in CLL^58^) is the subject of ongoing clinical trials.^59^ However, the often severe and occasionally life-threatening toxicities associated with lenalidomide may limit deliverability of such combinations, particularly for frail or elderly patients.^60^ In addition, not all therapeutically active anti-CLL agents are suitable for combination immunotherapy; for example, ibrutinib can actually inhibit NK function^61^ and may not be appropriate for combination with antibody therapies. Reovirus, in contrast, offers a means of augmenting humoral immunotherapy with minimal toxicity, and would be best utilised to treat minimal residual disease and/or recently relapsed patients.^62^

This study demonstrates that (i) CLL cells are susceptible to both direct reovirus-induced oncolysis and reovirus-enhanced NK cell killing, (ii) reovirus activates NK cells in CLL patient samples via an IFNα- and CD14^+^ monocyte-dependent mechanism, and (iii) reovirus together with anti-CD20 antibodies represents a promising combination strategy for the treatment of CLL. Despite improvements in therapy, the need remains for targeted, effective, deliverable and minimally toxic therapies for this incurable malignancy: reovirus in combination with anti-CD20 immunotherapy is a rational strategy that satisfies all these requirements and now warrants clinical evaluation.

## Figures and Tables

**Table 1:** Clinical characteristics of patients donating samples

	*Direct killing assays (*n*=9)*^a^	*Degranulation assays (*n*=24)*	*Interferon-α production (*n*=12)*
*Demographics*			
Age: median (range) (years)	62 (50–76)	71 (50–85)	72 (54–85)
Gender			
Male	6	15	6
Female	3	9	6
			
*Peripheral blood cell counts*			
Haemoglobin: median (range) (g/dl)	144 (101–158)	141 (93–163)	143.5 (130–163)
Platelets: median (range) (×10^9^/l)	202 (118–570)	178.5 (74–308)	186.5 (131–308)
White cell count: median (range) (×10^9^/^ ^l)	56.9 (20.7–145.0)	17.6 (8.9–125.1)	17.4 (8.9–34.9)
Lymphocyte count: median (range) (×10^9^/ l)	51.1 (17.4–138)	12.4 (4.7–112.7)	186.5 (131–308)
Monocyte count: median (range) (×10^9^/^ ^l)	0.51 (0.08–1.14)	0.47 (0.08–2.48)	0.5 (0.08–0.83)
			
*CLL stage and prior therapy*			
Stage			
Binet			
A	7	19	12
B	2	3	0
C	0	2	0
Rai			
Low (0)	6	18	11
Intermediate (1–2)	1	4	1
High (3–4)	2	2	0
Prior lines of therapy: median (range)	0 (0–1)	0 (0–3)	0 (0–1)
Prior therapy with			
Fludarabine	0	1	0
Cyclophosphamide	0	1	0
Alemtuzumab	0	2	1
Rituximab	0	1	0
Corticosteroid monotherapy	1	0	0
Median time from last therapy (months) (for previously treated patients)	28	30	30
			
*CLL phenotype*			
CD20 expression			
Absent	1	2	0
Weak	9	15	8
Present	0	7	4
IgH mutational status			
Unmutated	1	2	1
Mutated	5	8	4
Unknown	3	14	7
TP53 status			
Wild type	6	12	7
Mutated	1	1	0
Unknown	2	11	5
ATM status			
Wild type	5	13	7
Mutated	2	0	0
Unknown	2	11	5
Other cytogenetic abnormalities			
Del 13q14	0	5	2
Add 12	0	1	0
Normal	5	8	5
Unknown	2	8	5

Abbreviations: ATM, ataxia telangiectasia mutated; CLL, chronic lymphocytic leukaemia; IgH, immunoglobulin H.

Surface expression of CD20 was determined by flow cytometry and *TP53*, *ATM* and other cytogenetic abnormalities by interphase fluorescence *in situ* hybridisation using the Vysis LSI CLL FISH Probe Kit (Abbott Molecular Inc., Abbott Park, IL, USA).

a Additional clinical data were unavailable for one sample.

## Notes

Matt Coffey is an employee of Oncolytics Biotech Inc., with company stock and options. All the other authors declare no conflict of interest.
