# Supplementary material for: Oncolytic reovirus enhances rituximab-mediated antibody-dependent cellular cytotoxicity against chronic lymphocytic leukaemia
Source: Leukemia. 2015 Apr 24;29(9):1799–810. doi: 10.1038/leu.2015.88 (PMC4490165; doi:10.1038/leu.2015.88)
Supplement: Supplementary Figures [file leu201588x1.doc]

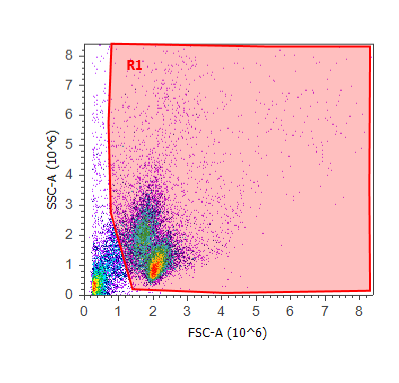

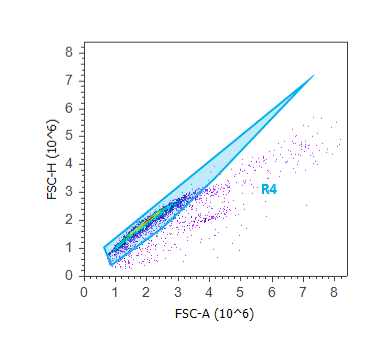

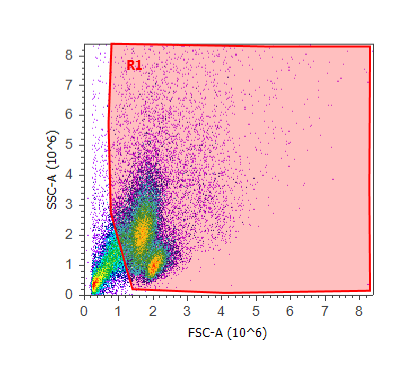

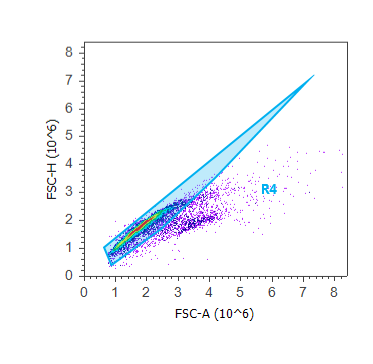


Intact cell gate

Excluding doublets

**No Virus**

Excluding doublets

Intact cell gate

**10 PFU/cell Reovirus**


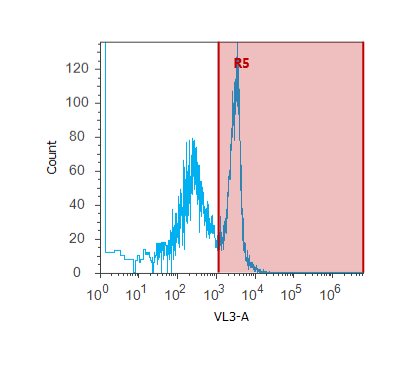

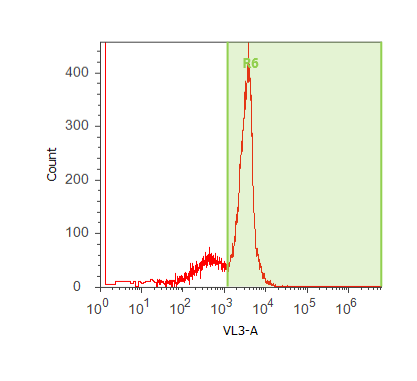

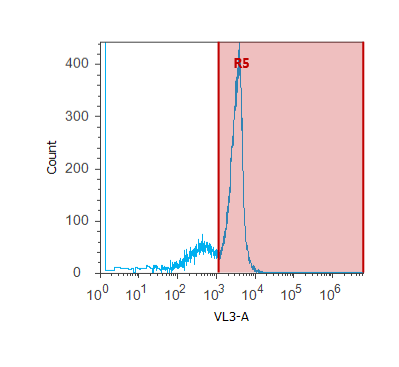


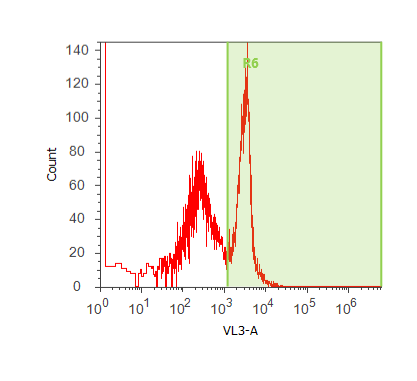


40.8%

40.5%

72.7%

72.3%

Percentage of dead cells

**Supplementary Figure 1: Exclusion of cell aggregates/doublets does not influence reovirus-induced cytotoxicity data.** CLL cells were either left untreated (no virus), or treated with 10pfu/cell reovirus, for 7 days prior to PI staining and flow cytometry analysis. Cell viability excluding cell debris (intact cell gate) or excluding cell aggregates/doublet was determined; levels of cell death (PI^+^ cells) were comparable using both analysis methods.

**
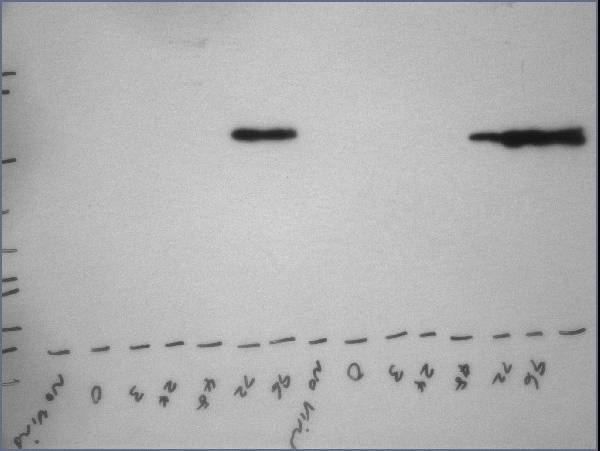

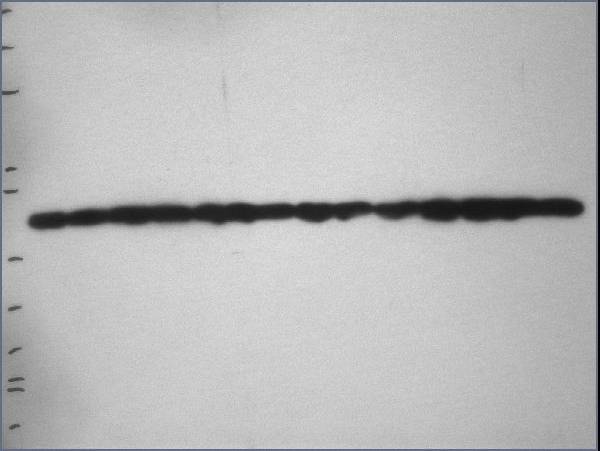
**

No Virus

No Virus

σ3

GAPDH

0

3

24

72

96

Time (hrs)

**MEC2**

**EHEB**

48

0

3

24

48

72

96

**Supplementary Figure 2: Western blot for the reovirus sigma 3 protein in EHEB and MEC-2 cells.** EHEB and MEC-2 cells were either left untreated (no virus) or treated with 1pfu/cell reovirus for 0, 3, 24, 48, 72 and 96 hours. Cells were harvested and western blot cell lysates were made using RIPA buffer. 30ug of cell lysate was loaded onto a 10% SDS page polyacrylamide gel and proteins were separated by gel electrophoresis. Proteins were transferred to a PVDF membrane and expression of anti-reovirus sigma 3 was determined (Developmental Studies Hybridoma Bank; 4F2 (1:200). GAPDH was used as a loading control.

**
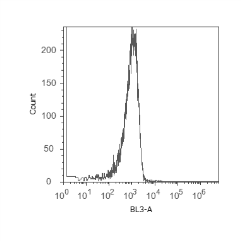

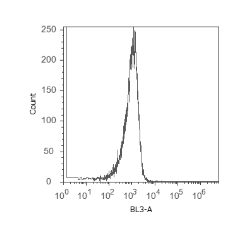

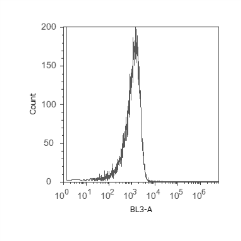

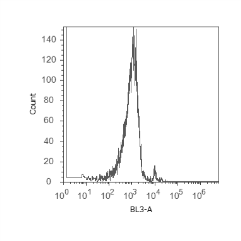

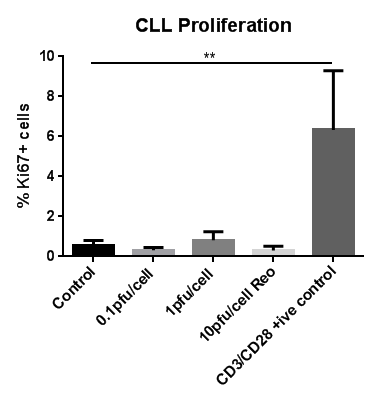
**

Ki67

0pfu/cell 1pfu/cell

10pfu/cell CD3/CD28

**Supplementary Figure 3: Reovirus does not stimulate CLL proliferation.** Primary CLL cells were treated with reovirus or anti-CD3/CD28 micro-beads (as a positive control) and the percentage of Ki67^+^ cells was determined by flow cytometry. Representative histogram plots are shown along with a bar chart showing the mean percentage of Ki67^+^ cells (+SEM; n≥3).

i)

ii)

**Supplementary Figure 4: Recombinant IFNα increases NK cell activation.** CLL patient PBMCs were treated with 5000pg/ml recombinant IFNαcells overnight and NK cell CD69 expression and CD107 degranulation was examined. Bar charts show i) the mean fluorescence intensity for CD69 expression and ii) the percentage of NK cell expressing CD107a/b in the presence or absence of EHEB/MEC-2 cell targets. Data shows the mean of n=4 patient samples.

A.)

B.)

**Supplementary Figure 5: Efficacy of reovirus and rituximab treatment using healthy donor PBMCs.**  Healthy donor PBMCs were either left untreated or cultured overnight with reovirus. A) 4hr ^51^Cr release assays were carried out using healthy donor PBMCs that were either left untreated or activated with reovirus overnight and co-cultured with rituximab-labelled targets. Line graphs compares the mean % lysis (n=4± SEM;). B) NK cell (CD3^-^CD56^+^) CD107a/b degranulation was determined after co-culture with rituximab-labelled or isotype control EHEB or MEC-2 cell targets. Bar charts show the mean percentage of total NK cells expressing CD107a/b (n=3, +SEM).

**Supplementary Figure 6: Potentiation of rituximab-mediated ADCC by replication-competent reovirus, UV-inactivated reovirus and recombinant IFNα.**  CLL patient PBMCs were either left untreated or cultured overnight with replication-competent (live reo) or UV-inactivated reovirus (UV-reo), or 5000pg/ml recombinant IFNα (IFNa). EHEB CLL cell targets were labelled with rituximab and co-cultured with untreated PBMCs (Rit Alone), or PBMCs that had been treated overnight (as above), for 4-5hrs. ^51^Cr release or cell tracker labelled/propidium iodide (PI) cytotoxicity assays were used to determine the percentage of dead EHEB cell targets. Fold increase in EHEB cell death, compared to untreated PBMCs (Rit Alone), was quantified and data shown is the mean of n=2 experiments (+SD).


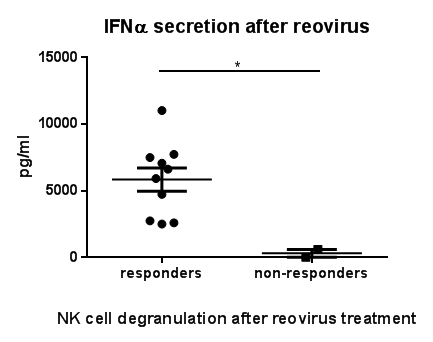


**Supplementary Figure 7: IFNα production is associated with reovirus-induced NK cell activation.** IFNα secretion was determined by ELISA and a comparison was made between patient PBMCs (n=12 samples) which demonstrated NK cell activation in response to reovirus (responders) and those which were not activated by reovirus (non-responders).
